# Dietary Modulations in Preventing Cardiometabolic Risk in Individuals with Type 2 Diabetes

**DOI:** 10.1007/s13668-024-00541-z

**Published:** 2024-05-20

**Authors:** Nursel Dal, Saniye Bilici

**Affiliations:** 1 Department of Nutrition and Dietetics, Bandirma Onyedi Eylul University, Balikesir, Turkey; 2 Department of Nutrition and Dietetics, Gazi University, Ankara, Turkey

**Keywords:** Type 2 diabetes, Cardiometabolic risk, Dietary modulations, Mediterranean diet

## Abstract

**Purpose of Review:**

Type 2 diabetes mellitus (T2DM) is a complex health issue include obesity, high cholesterol, high blood pressure, and chronic inflammation that increase the risk of cardiovascular diseases (CVDs). CVDs are of great concern in the disease progression and prognosis of T2DM. This review is a comprehensive examination of the literature on the relationship between T2DM and cardiovascular risk, nutrition-related cardiometabolic risk (CMR) factors, and impact of dietary modulations on CMR.

**Recent Findings:**

In recent years the researches has been focus on the importance of a comprehensive treatment approach like dietary modulations to address multiple cardiovascular risk reductions, including hypertension and dyslipidemia. Modulation of dietary patterns are the most promising interventions to prevent CMR factors and T2DM via affecting the body weight, glucose control, and microbial diversity of individuals. Current evidence suggests that high-quality dietary patterns such as the Dietary Approaches to Stop Hypertension (DASH) eating plan and the Mediterranean diet is important in the metabolic control processes of T2DM with anti-inflammatory and antioxidant compounds, glucagon-like peptide agonist compounds, and intestinal microbiota changes.

**Summary:**

Nutrition plays a critical role in preventing and improving CVD outcomes in patients with T2DM. Dietary modulations should be planned considering individual differences in responses to dietary composition and nutritional changes, personal preferences, eating behaviors and gut microbiota differences.

## Introduction

Type 2 Diabetes Mellitus (T2DM), an important public health problem worldwide, is a chronic metabolic disorder that results in hyperglycemia that develops due to impaired insulin secretion by the β-cells of the pancreas or the failure of insulin-sensitive tissues to respond to insulin [1]. Although it is known as adult-onset diabetes, it is associated with acute and chronic complications that cause a major impact on survival and quality of life, especially in patients diagnosed at a younger age [2].

Associated with the increasing burden of obesity, T2DM is expected to affect more than 600 million individuals worldwide in the next 20 years [3]. According to World Health Organization (WHO) estimates, approximately 5 million people die every year due to mismanagement of diabetes, and by 2030, diabetes is predicted to be the seventh leading cause of death globally [1]. Therefore, preventive actions are needed to reduce the worsening of the prognosis of the disease and associated complications. Steps to reverse excess body weight, unhealthy or wrong dietary practices, and a sedentary lifestyle are some of the main points in treating the disease and reducing complications [4]. Recent studies point out that the consumption of foods high in total fat, saturated fat, and cholesterol, accompanying eating disorders, and changes in the intestinal microbiota regard with the dietary pattern of individuals with T2DM increase the cardiometabolic risk [5–7]. Among many modifiable factors that contribute to the development of cardiovasculer diseases (CVDs) with T2DM, diet plays a critical role, and a healthy diet is associated with improved cardiometabolic health.

The term cardiometabolic risk (CMR) refers to clinical abnormalities, include hyperinsulinaemia, abdominal obesity, atherogenic dyslipidaemia and elevated blood pressure, that predict chronic disease such as CVDs and/or T2DM [8, 9]. Weight loss represents the main therapeutic goal to treat obesity and prevent CMR factors and T2DM. Rather than the long-term maintenance of weight reduction following restricted calorie diets, changes in diet composition acting on nutrient quality independently of changes in energy intake may be effective in cardiometabolic and T2DM risk prevention, offering a more feasible alternative treatment to energy restriction. Greater adherence to dietary patterns and consumption of dietary components linked to a preventive effect on CMR factors, T2DM, or other chronic diseases, are two characteristics of higher diet quality [10–12].

Whilst dietary interventions have been shown to reduce obesity and the risk of T2DM, specific information regarding dietary composition is often lacking. In this line, the literature search was conducted focusing on the last 10 years, between 2013 and 2023, using electronic databases (PubMed, Web of Science, and Google databases Embase and MEDLINE) for all available publications in English. Search terms included T2DM, CMR, inflammation, obesity, dietary habits, dietary patterns and dietary modulations. This narrative review explores available data about the relationship between T2DM and cardiovascular risk, nutrition-related CMR factors, and impact of dietary modulations on CMR.

## T2DM and Cardiovascular Risk

It is stated that individuals with T2DM have cardiovascular risk factors, and this comorbid condition continues to be an important public health problem with clinical and socioeconomic implications. The major CVDs associated with T2DM include ischemic heart disease, heart failure, stroke, coronary artery disease, and peripheral artery disease, and these complications can result in death for at least 50% of patients with T2DM. T2DM is characterized by insulin resistance, which is associated with a higher relative risk of cardiovascular events, is usually accompanied by abnormal lipid metabolism. Elevated blood glucose is strongly associated with the risk of both macrovascular and microvascular complications in patients with T2DM [13]. Hyperglycemia contributes to myocardial damage, ischemic events, and thrombosis, and may ultimately cause vascular dysfunction. Adverse cardiovascular outcomes such as atherosclerotic CVDs and heart failure are the main causes of morbidity and mortality in T2DM [14]. In addition to failure to control glucose, insulin resistance, obesity, hypertension, dyslipidemia, poor eating habits, physical inactivity, and smoking are factors that increase the risk of CVDs [15].

## Nutrition-Related Cardiometabolic Risk Factors

**Fig. 1:**
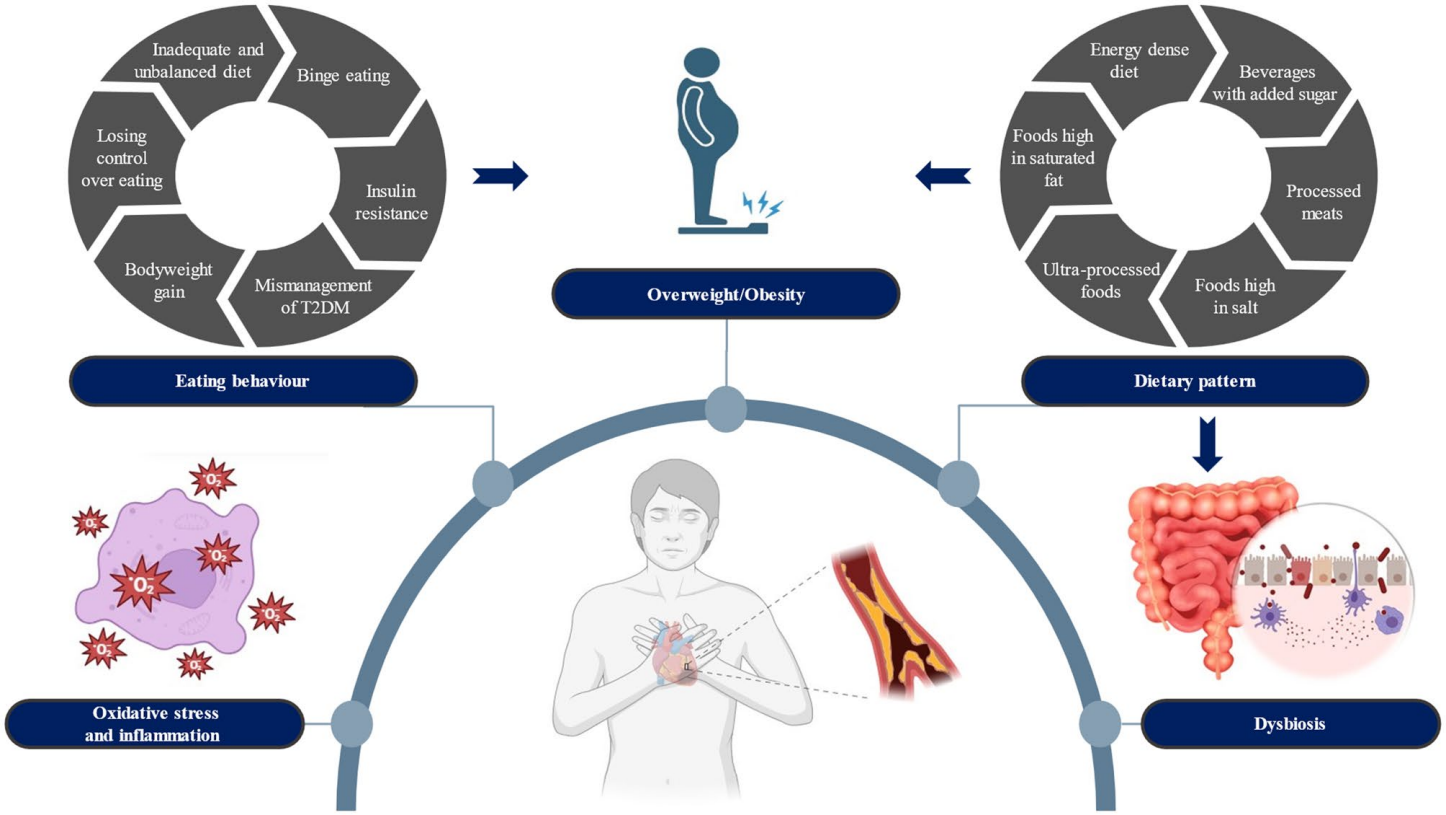
Nutrition-related cardiometabolic risk factors in T2DM

The metabolic effects of diet have been associated with long-term CMR, independent of lifestyle (such as physical inactivity, smoking, and alcohol consumption) and traditional risk factors. In this context, several nutrition-related mechanisms have been reported, reflecting the interactions between individuals' microbiome characteristics, nutritional components, and metabolites and the pathways that cause CMR [16••]. It is stated that individuals' eating behavior affects food intake, dietary patterns, and body weight and that this situation creates changes in microbial diversity, affecting metabolic control in individuals with T2DM and paving the way for the formation of comorbidities [6, 16••, 17]. Nutrition-related CMR factors in T2DM are summarized in Fig. 1.

The importance of nutrition in preventing CVDs and T2DM individually is well established in several studies that focused on macronutrients (carbohydrates, fats, proteins, macrominerals), micronutrients (vitamins and other minerals), and other nutrients (fiber, and dietary supplements) to control the balance between energy expenditure and calorie intake. It is necessary to highlight the importance of food quality over food quantity by having dietary patterns rich in whole grains, fruit, vegetables, nuts, legumes, fish, or vegetable oils and poor in processed meats, refined grains, refined carbohydrates, and salt [18–21]. An energy-dense dieatry pattern high in saturated fat and free sugar causes the temporary emergence of some metabolic and physiological derangements or dysfunctions, including oxidative stress, low-grade inflammation, and endothelial dysfunction, which act directly on postprandial plasma triglycerides and glucose. Low-grade inflammation and endothelial dysfunction, when combined with insulin resistance, a third risk factor for T2DM and CVD, increase CMR [22].

It is thought that the imbalance in the plasma of dietary fatty acids, especially the ratio of saturated fatty acids to polyunsaturated fatty acids, is effective in the development of CMR. It is stated that the ratio of polyunsaturated fatty acids, especially n-3 fatty acids, modulates CMR, inflammatory status, and atherogenic biomarkers. Therefore, the pattern of fatty acids provided in the diet is very important [23].

The increase in plasma-free fatty acid levels, especially saturated fatty acids, and the decrease in unsaturated fatty acids play an important role in the development of insulin resistance by inhibiting carbohydrate oxidation, which increases risk of T2DM [24]. The imbalance between saturated fatty acids and polyunsaturated fatty acids in the composition of dietary fats is positively associated with various metabolic disorders characterized by inadequate insulin function in non-glucose-dependent tissues (skeletal muscle, liver, and adipose tissue) and other abnormalities such as chronic inflammation, pancreatic cell loss, and atherosclerosis [25]. In addition, atherothrombotic complications caused by lipids can lead to endothelial dysfunction and contribute to other disorders related to plasma homocysteine and lipoprotein (Lp(a)) levels [24]. On the other hand, the alpha-linolenic acid (C18:3 n3) / linoleic acid (C18:2 n6) ratio and the docosahexaenoic acid (DHA)/eicosapentaenoic acid (EPA) ratio, which are polyunsaturated fatty acids, provide a positive vascular effect against the development of atherosclerosis, mostly in T2DM. EPA and DHA increase high-density lipoprotein (HDL) cholesterol by causing hypotriglyceridemic effects by inhibiting the hepatic synthesis of very low-density lipoproteins (VLDL) and help reduce cholesterol through increased apolipoproteinemia A1 synthesis [23].

Different foods, specifically those with high density energy but low nutritional value, have been associated with an increase in CMR factors. Mounting evidence relates CVDs and CMR factors to the consumption of ultra-processed foods [26]. The consumption of ultra-processed foods has dramatically increased in the last decades [27, 28] and is associated with lower diet quality and a high consumption of free sugars, total and saturated fats and lower consumption of fibre, proteins, and several minerals and vitamins. Notably, findings from a French prospective study reveal that high ultra-processed foods consumption is linked to a 12% higher risk of CVDs [29] and a 15% higher risk of diabetes after 5–6 years [30]. Similarly, a prospective study at Navarra University (SUN) associates the highest ultra-processed foods consumption with a 21% higher risk of hypertension after a 9-year follow-up [31]. These observations highlight potential health risks associated with increased intake of ultra-processed foods [29–31].

Obesity especially in childhood and adolescence is a cardiovascular risk factor for great impact, as it predisposes individuals to associated comorbidities, such as arterial hypertension, dyslipidaemia, and diabetes, all of which are implicated in an increased risk of cardiovascular events. Identification of obesity should be associated with the investigation of its origin, which may be related to poor eating habits, ingestion of foods of low nutritional value, or inadequate eating behaviour related to emotional factors [32].

Eating behavior is an important issue regarding T2DM treatment and is very effective in glycemic control. Binge eating behavior and related eating disorders are especially common in individuals with T2DM. In a case–control study examining the effect of diabetes on the risk of binge eating disorder, binge eating syndrome in individuals with T2DM was found to be 14% in diabetic individuals and 4% in the healthy control group. Recent studies also show that the prevalence of eating disorder behaviors, especially binge eating disorder, increases in patients with T2DM [33, 34]. Eating psychopathology is frequently observed in individuals with T2DM, and the presence of a comorbid eating disorder is associated with poorer glycemic control in individuals with a higher body mass index (BMI). Therefore, studies recommend routine evaluation of eating psychopathology in T2DM patients for both weight management and glycemic control [7, 33].

Papelbaum et al. [7] reported that 20% of patients with T2DM have an eating disorder, predominantly binge eating disorder. The authors reported that patients with eating disorders (32.6 ± 4.8 kg/m^2^) had higher BMI than patients without eating disorders (30.0 ± 5.2 kg/m^2^). Poorer glycemic control was found in patients with eating disorders compared to those with normal eating behaviors, regardless of age or duration of diabetes (p < 0.05). It has been reported that body weight may play an important role in modulating the relationship between eating disorders and glycemic control in individuals with T2DM [7].

In recent years, interest in the microbiota connection between the gut and the cardiovascular system has increased significantly, with the discovery that gut microbiota-derived molecules contribute to the development and risk factors of CVDs [6]. Nutrition is especially important for changing the intestinal microbiota. High total fat and saturated fat combined with low dietary fiber in Western-style nutrition negatively affect intestinal permeability and microbiota [35]. Trimethylamine (TMA), which is formed by the metabolism of choline and L-carnitine found in animal sources such as red meat, milk and dairy products, poultry, fish, and eggs by intestinal microorganisms, is oxidized to trimethylamine-N-oxide (TMAO) in the liver [36]. Recent studies show that high plasma TMAO levels in individuals with T2DM may be a new risk factor for CVD [37–39]. In a study conducted on CMR pathways with circulating metabolites and food groups, TMAO was found to potentially interact metabolically (p < 0.05) with host traits associated with CMR (1.55-fold) and could be modulated by plant-based nutrition [16••].

Similarly, it has been reported that there is an increase in serum zonulin levels, which can modulate intestinal permeability, in the presence of increased waist circumference, insulin resistance, dyslipidemia, inflammation-related coronary heart disease, and T2DM [5, 40]. In a study examining serum parameters for complications of diabetes in individuals with diabetes (n = 90), it was determined that serum zonulin levels showed a positive correlation with total cholesterol, LDL cholesterol, triglyceride, and HOMA-IR levels (p < 0.001) [41]. It has been reported that serum intestinal fatty acid binding protein (I-FABP/FABP2), which is an intracellular protein expressed in intestinal epithelial cells and functions to bind and transport fatty acids, causes atherosclerotic plaque formation in macrophages by affecting lipid and inflammatory responses. In addition, it has been stated that serum I-FABP levels may indicate the presence of obesity, insulin resistance, and T2DM and may be associated with cardiovascular risk [42]. Additionally, serum I-FABP level was found to be positively associated with the duration of hyperglycemia in patients with different diabetes courses (158 outpatients and 122 inpatients) (β = 0.362, p < 0.001) [43].

## Dietary Modulations for Reducing CMR

Lifelong medical nutrition therapy, along with medical treatment, is very important in the management of T2DM. Lifestyle changes, including diet and exercise, have always been the cornerstone of managing T2DM. Medical nutrition therapy provides a positive effect on improving glycemic control and metabolic outcomes by modulating the nutrition of patients. In particular, a 5–10% loss of body weight within six months is stated as a therapeutic target for both glycemic and metabolic control [44].

Restrictive hypocaloric diet practices targeting body weight loss, although providing rapid results in the early stages, subsequently lead to the regain of recorded body weight in most individuals and reduce the possible beneficial effect on CMR factors. At this point, changes in diet, independent of body weight loss, may reduce subclinical cardiac damage and inflammation in parallel with the reduction of CVD risk factors. Focusing on several important changes in terms of diet quality, such as increasing the consumption of whole grain products, legumes and nuts, vegetables, fruits, fish, milk and yogurt, and extra virgin olive oil instead of refined grain products, red meat, and processed meat, may have a positive effect on cardiometabolic health [10]. A recent meta-analysis (n = 13) found that although healthy eating programs did not have significant identified effects on CMR factors such as blood pressure and lipid profile, individuals increased their diet quality, fruit and vegetable consumption (22% increase), BMI (95% CI: 0.2–1.1; I^2^ = 6.4%), and HbA1c (95% CI: 0.1–1.6; I^2^ = 92%) [45]. A meta-analysis of 25 prospective cohort studies (3.8–25.0 years) examining the impact of dietary patterns on the development of T2DM indicates that higher consumption of red meat, processed meat, french fries, and refined grains, which are high in total fat and saturated fat content with characterized dietary patterns, is associated with a higher incidence of T2DM (IRR = 1.104, 95% CI: 1.059–1.151) [46]. Considering the potential cardiometabolic effects of consuming foods high in total fat, saturated fat, and cholesterol, therapeutic dietary modification is recommended as part of the American National Cholesterol Education Program Adult Treatment Panel Guidelines for individuals eating diets rich in these nutrients. Individuals' adherence to the diets called Step 1 and Step 2 is measured by MEDFICTS (Meats, Eggs, Dairy, Fried foods, fat in baked goods, Convenience foods, fats added at the Table, and Snacks), a nutrition assessment tool developed within the scope of the American National Cholesterol Education Program. Nutritional status can be evaluated in terms of heart health. The Step 1 diet includes < 30% of daily energy intake from total fat, < 10% from saturated fat, and < 300 mg of dietary cholesterol intake. The Step 2 diet includes < 30% of daily energy intake from total fat, < 7% from saturated fat, and < 200 mg of dietary cholesterol intake per day [5].

American Diabetes Association recommendations support a variety of healthy dietary approaches to achieve glycemic control and body weight management, but the effects of dietary interventions on cardiovascular outcomes in individuals with T2DM have not been widely studied [47]. Although there are a few studies indicating that the Dietary Approaches to Prevention of Hypertension (DASH) diet may be beneficial in preventing and managing the risk of CVDs in individuals with diabetes, it has not yet been clarified [48, 49]. Table 1 shows dietary intervention studies in recent years aimed at preventing cardiovascular risk in individuals with T2DM. In this context, randomized controlled studies that do not include experimental rat studies, are conducted only with humans and include dietary interventions are presented [48, 50, 51, 52•, 53]. Low-carbohydrate diets, ketogenic diets, Paleolithic diets, and high-protein and vegetarian diets are considered dietary approaches in T2DM management. Considering the sustainability of these diets, the Mediterranean diet is stated to be the nutritional model that provides the most effective benefit to glycemic control and body weight loss in T2DM [50, 51, 52•, 53]. In addition to these data, for the first time, the PREDIMED study firmly indicated that the Mediterranean diet reduced the risk of developing type 2 diabetes by 52% in patients who had no diabetes at the beginning of the study [54–56].

**Table 1:** Dietary interventions for modulation of cardiovascular risk in individuals with T2DM

**Sample characteristics**	**Nutritional intervention**	**Intervention duration**	**Results**	Reference
31 patients with T2DM with a mean age of 55.0 ± 6.5 years and diabetes duration of 4.0 ± 0.5 years	Each patient was given a control diet (50–60% carbohydrates, 15–20% protein, < 30% total fat, and < 5% simple carbohydrates) and a DASH diet	8 weeks in total, including 4 weeks of the control diet and 4 weeks of the DASH diet	Fasting plasma glucose level following the DASH diet compared to the control diet was 13.9 ± 4.5%, waist circumference 5.6 ± 1.2% and body weight 5.9 ± 1.1%, LDL cholesterol level 7.7 ± 3.3%, systolic and diastolic blood pressure decreased by 9.6 ± 1.8% and 9.9 ± 3.6%, respectively	[48]
Patients with T2DM, aged 18 and over, with a BMI value of ≥ 25 kg/m^2^	Control group (n = 117) Intervention group (n = 94): Low-fat vegan diet (vegetable oil intake < 3 g/serving/day)	18 weeks	Total cholesterol and LDL cholesterol levels decreased by 13.7 and 13.0 mg/dL in the intervention group and by 1.3 and 1.7 mg/dL in the control group (p < 0.001). HbA1C decreased by 0.7% and 0.1% in the intervention and control groups, respectively (p < 0.01)	[50]
108 patients with T2DM with a mean age of 60 ± 10 years, diabetes duration of 11 ± 7 years, and BMI value of 35.2 ± 7.7 kg/m^2^	A healthy nutrition program (40–45% low glycemic index carbohydrates, 1–1.5 g/kg/day protein, < 10% saturated fat, < 2300 mg/day sodium and 14 g/1000 kcal fiber)	16 weeks A group (n = 36): only dietician meeting B group (n = 36): dietician meeting and nutritional program C group (n = 36): dietician meeting, nutritional program and phone support	HbA1c value decreased significantly in groups B and C, except in group A (- 0.66%, 95% CI: -1.03,—0.30 and -0.61%, 95% CI: -1.0, -0.23 respectively) There was a significant decrease in body weight, body fat percentage, and waist circumference in Groups B and C	[51]
33 prediabetic or T2DM patients, aged 41 to 77 years (median 60.5 years), with BMI between 22.7 and 39.7 kg/m^2^	The ketogenic diet (20–50 g/day carbohydrate, ~ 1.5 g/kg protein) and Mediterranean diet for each patient	24 weeks in total, including 12 weeks of ketogenic diet and 12 weeks of Mediterranean diet	As a result of both diets, there was an improvement in HbA1c level (ketogenic diet -9%, Mediterranean diet -7%). The ketogenic diet provided a significant improvement in triglyceride levels (p = 0.02), and the Mediterranean diet provided a significant improvement in LDL cholesterol levels (p = 0.01)	[52]
228 patients with T2DM with a mean age of 57.3 ± 9.28 years and diabetes duration of 9.8 years	Control group (n = 123) Intervention group (n = 105) Mediterranean diet training and Mediterranean diet treatment by a nutritionist	6 months	HbA1c level decreased statistically in the intervention group compared to the control group (p < 0.001). An improvement in LDL cholesterol, systolic and diastolic blood pressure was detected in the intervention group (p < 0.05)	[53]

### Mediterranean Diet

The traditional Mediterranean diet is a dietary model that is characterized by consuming seasonal and local products, including high consumption of fruits, vegetables, grains, legumes, and nuts; moderate consumption of fish and white meat; and low consumption of red meat; it is low in saturated fats and sustainable for health and ecosystems [57]. It is a leading dietary model in the management of T2DM, including anti-inflammatory and antioxidant compounds, glucagon-like peptide agonist compounds, and changes in the intestinal microbiota. Indeed, each component of the Mediterranean diet may be involved in processes related to diabetes homeostasis, many of which share common physio-pathological pathways [58]. Tosti et al. [57] summarized the most important effects of the foods and nutritional components in the Mediterranean diet on health as follows: (a) lipid-lowering effect, (b) protection against oxidative stress, inflammation, and platelet aggregation, (c) modification of hormones and growth factors involved in cancer pathogenesis, (d) inhibition of nutrient sensing pathways by specific amino acid restriction, and (e) gut microbiota-mediated metabolite production affecting metabolic health. Regarding these health impacts of Mediterranean diet, as a healthy and sustainable nutrition model, is mostly preferred in the prevention and treatment of many diseases.

A systematic review (n = 24) examining the effect of the Mediterranean diet on diabetes control and cardiovascular risk modification reported that adherence to the Mediterranean diet has a protective role on glycemic control, such as a reduction in HbA1c (OR = 0.9%, 95% CI: 0.5–1.2, p < 0.001) and lower fasting plasma glucose levels (-32.8 mg/dL, p < 0.001; -21.0 mg/dL, p < 0.01), in addition to reduced insulin resistance and mortality [59]. In a meta-analysis, it was stated that the low-carbohydrate diet, Paleolithic diet, vegetarian diet, and Mediterranean diet significantly reduced HbA1c (-0.82% to -0.47% decrease) and fasting plasma glucose (-1.61 to -1.00 mmol/L decrease) compared to the control diet, but the Mediterranean diet was the most effective nutritional approach to improving glycemic control in patients with T2DM [60]. Similarly, compared to a low-fat diet, the Mediterranean diet has been reported to improve glycemic control, body weight loss, and cardiovascular risk factors such as triglyceride, total cholesterol, and HDL cholesterol in individuals with T2DM [61]. In addition, adherence to the Mediterranean diet positively affected the gut microbiota and concentrations of metabolites such as TMAO [62]. It has been stated that most of the potential effects of the Mediterranean diet come from its bioactive component content, including various polyphenols and mono- and polyunsaturated fatty acids [63]. In addition to these effects, regular physical activity, which is important for heart health, is also a part of the Mediterranean lifestyle, which is affected by the climate [47].

## Conclusion

CVDs are the most common cause of morbidity and mortality in developed countries. The prevalence of CVDs is much higher in patients with T2DM, who may benefit from lifestyle changes, which include adapted diets. Considering the burden that both diabetes and its complications pose to healthcare services, identifying new strategies to monitor and control diabetes and better characterize its complications becomes an important clinical necessity. In particular, clinical control of individuals' eating psychopathology may improve adherence to nutritional recommendations and help reduce postprandial hyperglycemia and its risks. Modulating nutrition may be effective in preventing the occurrence of comorbidities by affecting individuals' body weight, glucose control, and microbial diversity. Current evidence suggests that healthy dietary patterns such as Mediterranean diet, plant‐based diets, etc. is associated with lower CVD and T2DM risk, and healthier cardiometabolic indices. Dietary interventions must continue tailoring diets to improve T2DM and CVD outcomes; however, the dietary modulations should planned considering individual differences in responses to dietary composition and nutritional changes, the personal preferences like tradition, culture, or religion, eating behaviors and the critical role of individual gut microbiota in the crosstalk between diet, CVDs, T2DM.
